# Comparative Analysis of Phytochemical Composition of Gamma-Irradiated Mutant Cultivars of *Chrysanthemum morifolium*

**DOI:** 10.3390/molecules24163003

**Published:** 2019-08-19

**Authors:** Jaihyunk Ryu, Bomi Nam, Bo-Ram Kim, Sang Hoon Kim, Yeong Deuk Jo, Joon-Woo Ahn, Jin-Baek Kim, Chang Hyun Jin, Ah-Reum Han

**Affiliations:** Advanced Radiation Technology Institute, Korea Atomic Energy Research Institute, Jeongeup-si, Jeollabuk-do 56212, Korea

**Keywords:** *Chrysanthemum morifolium*, asteraceae, gamma irradiation, flavonoid, phenolic acid, anthocyanin, carotenoid, volatile compound, HPLC-DAD-ESIMS, GC-MS

## Abstract

The flowers of chrysanthemum species are used as a herbal tea and in traditional medicine. In addition, members of the genus have been selected to develop horticultural cultivars of diverse floral colors and capitulum forms. In this research, we investigated the phytochemical composition of eight gamma-irradiation mutant cultivars of *Chrysanthemum morifolium* and their original cultivars. The mutant chrysanthemum cultivars were generated by treatment with various doses of ^60^Co gamma irradiation of stem cuttings of three commercial chrysanthemum cultivars as follows: ‘ARTI-Dark Chocolate’ (50Gy), ‘ARTI-Purple Lady’ (30 Gy), and ‘ARTI-Yellow Star’ (50 Gy) derived from ‘Noble Wine’; ‘ARTI-Red Star’ (50 Gy) and ‘ARTI-Rising Sun’ (30 Gy) from ‘Pinky’; ‘ARTI-Purple’ (40 Gy) and ‘ARTI-Queen’ (30 Gy) from ‘Argus’; and ‘ARTI-Rollypop’ (70 Gy) from ‘Plaisir d’amour’. Quantitative analysis of flavonoids, phenolic acids, anthocyanins, and carotenoids in the flowers of the 12 chrysanthemum cultivars was performed using high performance liquid chromatography-diode array detector-electrospray ionization mass spectrometry (HPLC-DAD-ESIMS). Essential oils from the flowers of these cultivars were analyzed by gas chromatography–mass spectrometry (GC-MS). The mutant cultivars, ‘ARTI-Dark Chocolate’, ‘ARTI-Purple Lady’, ‘ARTI-Purple’, and ‘ARTI-Queen’ showed higher total amounts of flavonoid and phenolic acid compared with those of the respective original cultivars. The mutant cultivars, ‘ARTI-Dark Chocolate’, ‘ARTI-Purple Lady’ and ‘ARTI-Purple’, which produce purple to pink petals, contained more than two-times higher amounts of anthocyanins compared with those of their original cultivars. Of the mutant cultivars, ‘ARTI-Yellow Star’ in which petal color was changed to yellow, showed the greatest accumulation of carotenoids. Ninety-nine volatile compounds were detected, of which hydrocarbons and terpenoids were abundant in all cultivars analyzed. This is the first report that demonstrated the phytochemical analysis of novel chrysanthemum cultivars derived from *C. morifolium* hydrid using HPLC-DAD-ESIMS and GC-MS. These findings suggest that the selected mutant chrysanthemum cultivars show potential as a functional source of phytochemicals associated with the abundance of health-beneficial components, as well as good source for horticulture and pigment industries.

## 1. Introduction

Chrysanthemum species belong to the Asteraceae family and comprise about 40 species distributed in Asia and Eastern Europe [1]. Chrysanthemum species are important as an ornamental and floricultural crop [2] and are among the most popular cut flowers in the horticulture industry owing to their high value as an ornamental plant, featuring various floral colors and spray types and uniformity of flowering time [3]. In addition, this flower also is used as a traditional medicine and a beverage in East Asia [4,5,6,7]. Phytochemicals of chrysanthemum species are important substances widely used for medicinal purposes because they show diverse bioactivities in therapeutic treatments. Chrysanthemum flowers effuse a strong aroma [7,8,9] and various compound types, carotenoids [10,11], anthocyanin [7,10], flavonoids [7,12,13], phenolic acids [7,12,13,14,15,16], and essential oils [17,18] have been identified from the chrysanthemum flower. These phytocemicals were found to have diverse biological activities such as antioxidant [7,12,15], neuroprotective [14], antiallergic [16] and antifungal activities [17].

**Figure 1:**
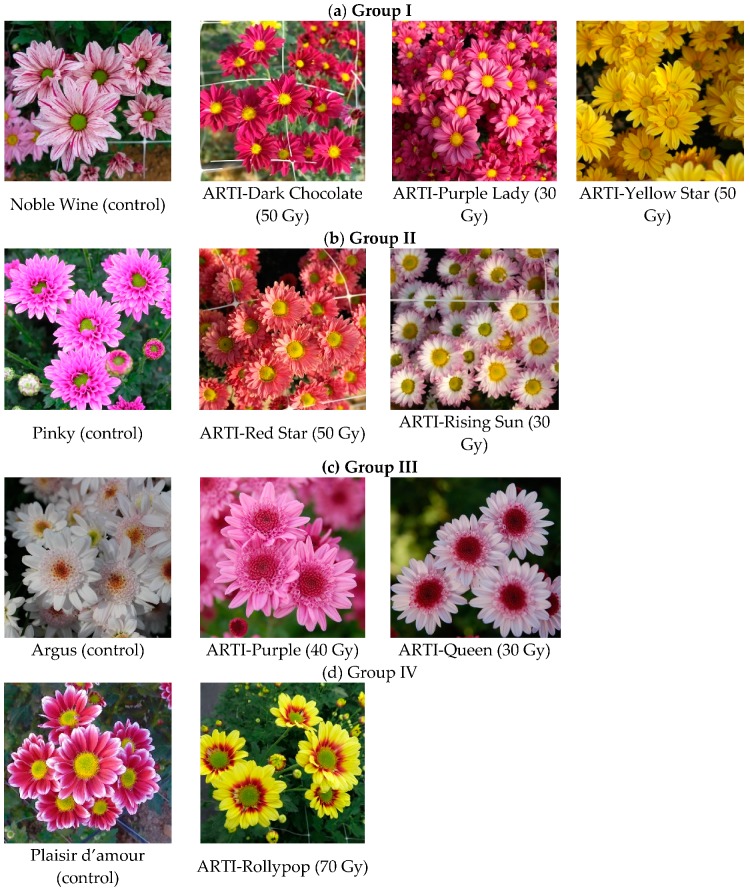
Twelve *Chrysanthemum morifolium* cultivars. (**a**) Group I: the mutant cultivars ‘ARTI-Purple Lady’ (30 Gy), ‘ARTI-Yellow Star’ (50 Gy), ‘ARTI-Dark Chocolate’ (50 Gy) derived from the original cultivar ‘Noble Wine’; (**b**) Group II: the mutant cultivars ‘ARTI-Red Star’ (50 Gy) and ‘ARTI-Rising Sun’ (30 Gy) derived from the original cultivar ‘Pinky’; (**c**) Group III: the mutant cultivars ‘ARTI-Purple’ (40 Gy) and ‘ARTI-Queen’ (30 Gy) derived from the original cultivar ‘Argus’; (**d**) Group IV: the mutant cultivars ‘ARTI-Rollypop’ (70 Gy) from the original cultivar ‘Plaisir d’amour’.

**Figure 2:**
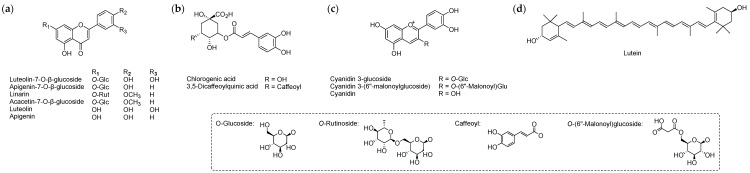
Structures of the standard materials. (**a**) Flavonoids; (**b**) phenolic acids; (**c**) anthocyanins; (**d**) carotenoids.

The cultivars of chrysanthemum species have been generated through hybridization, but the floriculture industry relies on a limited number of mutated traits established based on specific flower quality parameters and consumer palatability [19]. Mutation breeding involves the use of a mutagen to develop plants exhibiting novel mutated characteristics that do not disturb elite cultivar traits [20,21]. Novel chrysanthemum cultivars generated by radiation mutagenesis and showing improved agronomic characteristics have been developed using mutation breeding techniques [7,12,21]. Our research group has also developed novel chrysanthemum cultivars by gamma-irradiation on stem cuttings of the current available chrysanthemum cultivars and registered them in the Korea Seed and Variety Service (Figure 1) [22]. In our previous work, which explored the potential of a novel chrysanthemum cultivar as a functional food, we compared the active components among the ethanol extracts of the commercially available medicinal herb, *C. morifolium*, the original cultivar, ‘Novel Wine’, and the mutant cultivar, ‘ARTI-Dark Chocolate’ which is one of novel chrysanthemum cultivars developed by our research team [12]. In addition, we have also studied the component analysis, antioxidant activity evaluation, floral scent analysis for the hot water extracts of the commercially available chrysanthemum tea (cv. Gamguk) and the mutant cultivar, ‘ARTI-Dark Chocolate’ in order to develop a new chrysanthemum tea with improved quality and distinct color using this novel chrysanthemum cultivar [7]. Based on these prospective researches, we conducted the comparative analysis of phytochemicals of the flowers of all novel mutant cultivars of *C. morifolium* produced by our research team with those of their original cultivars, using the standard materials such as flavonoids, phenolic acids, anthocyanins and carotenoids (Figure 2), through the analysis of high performance liquid chromatography–diode array detector-electrospray ionization mass spectrometry (HPLC-DAD-ESIMS) and gas chromatography-mass spectrometry (GC-MS).

## 2. Results and Discussion

### 2.1. Analysis of Flavonoids and Phenolic Acids Contents

Flavonoids and phenolic acids in the flower of novel chrysanthemum cultivars were analyzed using HPLC-DAD-ESIMS. In their chromatograms (Figure S1), eight peaks were identified as chlorogenic acid (peak 1; 355 [M + H]^+^), luteolin-7-*O*-β-glucoside (peak 2; 449 [M + H]^+^), 3,5-dicaffeoylquinic acid (peak 3; 517 [M + H]^+^), apigenin-7-*O*-β-glucoside (peak 4; 433 [M + H]^+^), linarin (peak 5; 593 [M + H]^+^), acacetin-7-*O*-β-glucoside (peak 6; 447 [M + H]^+^), luteolin (peak 7; 287 [M + H]^+^), and apigenin (peak 8; 271 [M + H]^+^) by comparison of the retention time, ultraviolet (UV) spectra, and positive molecular ions with those of the compounds isolated or purchased in our previous study [12]. The contents of the eight compounds in the flower of novel Chrysanthemum cultivars were quantified using the method established by our previous study [12] (Table 1). A significant difference in the compound contents was observed between the original cultivar and those of the derivative gamma-irradiated mutant cultivars. In group I, ‘ARTI-Purple Lady’ exhibited a higher content of apigenin-7-*O*-β-glucoside, but lower or similar amounts of the other compounds, compared with those of the original cultivar (‘Noble Wine’). ‘ARTI-Yellow Star’ showed the highest content of acacetin-7-*O*-β-glucoside in group I, and 3,5-dicaffeoylquinic acid and luteolin contents in ‘ARTI-Yellow Star’ were slightly higher than those of ‘Noble Wine’. In our previous study on the analysis of the constituents of ‘Noble Wine’ and ‘ARTI-Dark Chocolate’ [12], the contents of 3,5-dicaffeoylquinic acid, luteolin, and apigenin were much higher than those of ‘Noble Wine’ and the contents of luteolin-7-*O*-β-glucoside and linarin were slightly higher. Linarin was detected only in group I cultivars. In group II, ‘ARTI-Red Star’ showed much lower amounts of flavonoid and phenolic compounds than those of the original cultivar (‘Pinky’), except for luteolin. ‘ARTI-Rising Sun’ exhibited contents of all compounds except for apigenin, which showed the highest content in group II, that were lower than or similar to those of ‘Pinky’. The content of 3,5-dicaffeoylquinic acid in ‘ARTI-Queen’ was the highest among the cultivars in group III; however, the contents of the other compounds were lower than or similar to those of ‘Argus’. ‘ARTI-Purple’ showed considerably higher amounts of luteolin and apigenin than those of the original cultivar ‘Argus’. Comparison of the original cultivar (‘Plaisir d’amour’) with that of the gamma-irradiated mutant cultivar (‘ARTI-Rollypop’) showed that linarin and acacetin-7-*O*-β-glucoside were not detected and the contents of all other compounds were similar in the two cultivars, except for luteolin and apigenin, for which higher contents were detected in ‘ARTI-Rollypop’ than those of the original cultivar. In each group, when the color of petals and/or petaloid disc was changed to purple or pink by radiation mutation breeding, the total amount of flavonoids and phenolic acids was more. It was assumed that the accumulation of flavonoids and phenolic acids was accelerated. On the other hand, the total amount of flavonoids and phenolic acids was less in the flowers in which the color of petals was changed to yellow, red, or white in each group. In the reports on the function of inside the plants, flavonoid glycosides were involved in protection against radiation stress and in producing a purple pigment [23]. In addition, the generation of caffeoylquinic acids was induced in an anthocyanin-accumulating plant cell line such as a purple sweet potato [24], and eventually the biosynthetic pathway of phenolic compounds can be closely related to that of anthocyanins [25]. Therefore, a color change of these γ-irradiated mutant chrysanthemum cultivars may be mediated by the accumulation of flavonoid glycosides and caffeoylquinic acids.

### 2.2. Analysis of Anthocyanin Contents

Anthocyanins were identified based on comparison of retention times and UV/Vis and mass spectra for the authentic standards, cyanidin-3-*O*-glucoside (Peak 1; 449 [M + H]^+^, cyanidin-3-*O*-(6″-malonylglucoside) (Peak 2; 535 [M + H]^+^), and cyaniding (Peak 3; 287 [M + H]^+^) (Figure S2). These compounds were also quantified in the original cultivars and the respective gamma-irradiation mutant cultivars using the method established by our previous study [7] (Table 2). In group I, ‘ARTI-Dark Chocolate’ (with dark purple petals) and ‘ARTI-Purple Lady’ (with pink petals) showed almost five-times and two-times higher contents of anthocyanins, respectively, than those of ‘Noble Wine’. Although anthocyanin content was comparatively analyzed between the hot water extract of commercially available chrysanthemum tea and the novel cultivar, ‘ARTI-Dark Chocolate’ in our previous study to develop a new chrysanthemum tea [7], the anthocyanin content analysis of ‘ARTI-Dark Chocolate’ was firstly compared to those of the original cultivar and other novel cultivars in this study. The contents of cyanidin-3-*O*-glucoside and cyanidin in ‘ARTI-Yellow Star’ (with yellow petals) were about two-times less than those in original cultivar and the content of cyanidin-3-*O*-(6″-malonylglucoside) was five-times lower in ‘ARTI-Yellow Star’ than that of the control plant. In the comparison between the original cultivar ‘Pinky’ (with bright pink petals) and ‘ARTI-Red Star’ (with light red petals) in group II, three anthocyanins were detected at slightly higher amounts in the mutant cultivar ‘ARTI-Red Star’. In contrast, anthocyanin content of ‘ARTI-Rising Sun’ (with white/light pink petals) were lower than those of ‘Pinky’. In group III, the gamma-irradiated mutants ‘ARTI-Purple’ (with purple petals and dark purple petaloid disc) and ‘ARTI-Queen’ (with light pink petals and dark purple petaloid disc) showed higher contents of anthocyanins than those of the control plant ‘Argus’ (with white petals and pale purple petaloid disc). In particular, the content of cyanidin-3-*O*-(6″-malonylglucoside) in ‘ARTI-Purple’ was three-times higher than that in ‘Argus’. ‘ARTI-Rollypop’ (with yellow/red petals) in group VI, which showed slightly higher contents of cyanidin-3-*O*-glucoside and cyanidin-3-*O*-(6″-malonylglucoside) and slightly lower content of cyanidin compared with those of the control plant ‘Plaisir d’amour’ (with pink/white petals). Therefore, these results suggested that the pink, purple, or red colored chrysanthemum flowers of the chrysanthemum cultivars, such as ‘ARTI-Dark Chocolate’, ‘ARTI-Purple Lady’, ‘ARTI-Red Star’, and ‘ARTI-Purple’ contained more anthocyanin contents than the white or yellow colored chrysanthemum flowers. Thus, a mechanism study of flower color variation elucidated by gene function analysis and transcriptome sequencing in these γ-irradiated mutant chrysanthemum cultivars—which have been changed to dark-purple, pink, or red petals—will be scheduled.

### 2.3. Analysis of Carotenoid Contents

The carotenoids extracted from the flowers of the chrysanthemum cultivars were analyzed using HPLC-DAD-ESIMS. However, due to unsatisfactory identification of each peaks for carotenoids detected at a wavelength of 450 nm by ESIMS, the total content of carotenoids in the chrysanthemum cultivars was calculated according to the total peak area of HPLC chromatograms at 450 nm. The large peaks at *t*_R_ 2−4 min in their chromatograms showed maximum absorbance at 230 and 270 nm from DAD, indicating that they were not carotenoids. All peaks after *t*_R_ 5 min in their chromatograms confirmed to be carotenoid compounds, all of which had a maximum absorbance at 420, 450, or 470 nm from DAD. Therefore, the sum of area values of these peaks was substituted into the calibration of lutein to calculate the total carotenoid contents. As a result, ‘ARTI-Yellow Star’ with yellow petals showed much higher content of carotenoids (0.163 ± 0.027 mg/g, dry weight) than the control plant with white petals and purple stripes, ‘Noble Wine’ (0.079 ± 0.005 mg/g, dry weight). Among its other gamma irradiated mutant cultivars, ‘ARTI-Dark Chocolate’ with dark purple petal (0.065 ± 0.005 mg/g, dry weight) had a slightly low level of carotenoids and ‘ARTI-Purple Lady’ (0.083 ± 0.008 mg/g, dry weight) with bright purple petals exhibited a slightly high content of carotenoids, compared with that of ‘Noble Wine’. In group II, the gamma-irradiated mutant cultivar with bright red petals, ‘ARTI-Red Star’ (0.121 ± 0.021 mg/g, dry weight) contained more carotenoids contents than the control plant, ‘Pinky’ (0.094 ± 0.009 mg/g, dry weight), which in contrast to another mutant cultivar with white/light pink petals, ‘ARTI-Rising Sun’, showed a low level of carotenoids (0.076 ± 0.015 mg/g, dry weight). In group III, the control plant, ‘Argus’ and its mutant cultivars, ‘ARTI-Queen’ and ‘ARTI-Purple’ showed almost the same carotenoids contents, 0.055 ± 0.002 mg/g, dry weight, 0.054 ± 0.003 mg/g, dry weight and 0.056 ± 0.003 mg/g, dry weight, respectively. The mutant cultivar of ‘Plaisir d’amour’, ‘ARTI-Rollypop’ has yellow/red petals, and its carotenoids content (0.130 ± 0.030 mg/g, dry weight) was slightly higher than that of ‘Plaisir d’amour’ (0.103 ± 0.013 mg/g, dry weight). As a comprehensive result, the mutant cultivar that changed into yellow petals, ‘ARTI-Yellow Star’ had the most accumulation of carotenoids, and ‘ARTI-Rollypop’ in which part of petal changed to yellow/red and ‘ARTI-Red Star’ with red petals showed increased carotenoids compared to each control plant. In addition, in the chromatograms of the yellow flowers, ‘ARTI-Yellow Star’ and ‘ARTI-Rollypop’, a specific peak at *t*_R_ 6−8 min was shown, however this peak, corresponding to certain carotenoid, was not identified. ‘ARTI-Yellow Star’ with yellow petals accumulated carotenoids twice as much as other chrysanthemum cultivars. In the previous study on functional analysis of genes related with the accumulation of carotenoid in the biosynthesis pathway [26,27,28], the deletion of the carotenoid cleavage dioxygenase gene (*CmCCD4a*) may be responsible for the development of yellow petals of chrysanthemum.

### 2.4. Analysis of Volatile Compounds

**Figure 3:**
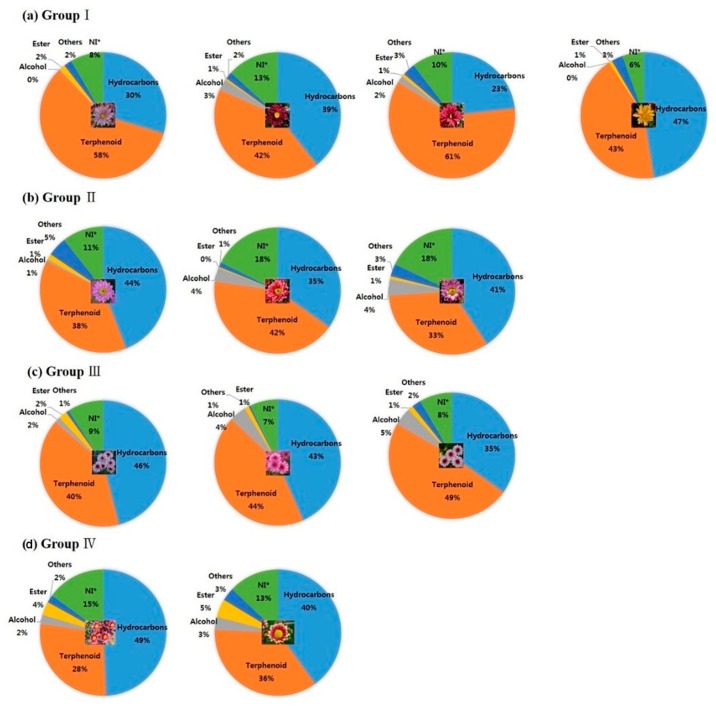
Relative contents (%) of volatile compounds released from the flowers of the 12 chrysanthemum cultivars. (**a**) Group I: ‘Noble Wine’, ‘ARTI-Dark Chocolate’, ‘ARTI-Purple Lady’, and ‘ARTI-Yellow Star’; (**b**) Group II: ‘Pinky’, ‘ARTI-Red Star’, and ‘ARTI-Rising Sun’; (**c**) Group III: ‘Argus’, ‘ARTI-Purple’, and ‘ARTI-Queen’; (**d**) Group IV: ‘Plaisir d’amour’ and ‘ARTI-Rollypop’. NI: not identified.

The volatile compounds identified in the flowers of the 12 chrysanthemum cultivars, together with their retention time, molecular formula, and contents (%) are shown in Table S1. Significant differences in volatile compound composition was observed among the chrysanthemum cultivars. The GC-MS analysis detected 99 volatile compounds in the novel chrysanthemum cultivars, of which 57 compounds were tentatively identified by mass spectra and retention time, based on a NIST library similarity index greater than 90%. The retention time of components may vary with individual chromatography systems, but the pattern of the gaps between retention times of ducane, eucalyptol, camphor, and bornyl acetate in the chemical profiling of the 12 chrysanthemum cultivars was similar to those of the literature [29]. In the present study, the retention index used to convert the retention time into a system-independent constant was not obtained. The volatile compounds were categorized as hydrocarbons, carboxylic acid, terpenoids, alcohols, esters, and not identified compounds (NI). Hydrocarbons and terpenoids were major volatile compounds present in all chrysanthemum cultivars (Figure 3). In group I, ‘ARTI-Purple Lady’ exhibited similar contents of volatile compounds to those of the original cultivar ‘Noble Wine’, while ‘ARTI-Dark Chocolate’ and ‘ARTI-Yellow Star’ showed higher contents of hydrocarbons and alcohols than those of ‘Noble Wine’ and ‘ARTI-Purple Lady’. In group II, ‘ARTI-Rising Sun’ exhibited similar in the distribution of volatile compounds of those of the original cultivar ‘Pinky’, whereas ‘ARTI-Red Star’ had a higher content of terpenoids and a lower content of hydrocarbons compared to those of ‘Pinky’ and ‘ARTI-Rising Sun’. In group III, ‘Argus’ and ‘ARTI-Purple’ showed similar contents of volatile compounds, however ‘ARTI-Queen’ exhibited higher amount of terpenoids and a lower content of hydrocarbons than those of its original cultivar, ‘Argus’. In group IV, ‘ARTI-Rollypop’ exhibited higher amount of terpenoids and a lower content of hydrocarbons compared to those of the original cultivar ‘Plaisir d’amour’.

Table 3 and Figure S4 summarizes the 10 most abundant constituents among volatile compounds detected in the GC-MS chromatograms of the 12 chrysanthemum cultivars. Among the top 10 volatile compounds of these chrysanthemum cultivars, three compounds, camphor, _DL_-α-tocopherol, and squalene—known as bioactive ingredients [30,31,32,33]—were found. Within the top 10 of volatile compounds, camphor showed its high content in ‘Noble Wine’, ‘ARTI-Purple Lady’, and ‘ARTI-Yellow Star’ of group I. Other chrysanthemum cultivars, ‘ARTI-Dark Chocolate’, ‘ARTI-Red Star’, ‘ARTI-Rising Sun’, ‘Argus’, and ‘ARTI-Queen’ contained this essential oil, but the content was less than 1.75% (Table S1). Squalene was found in all chrysanthemum cultivars except for ‘Pinky’ (Table S1). Especially, in ‘ARTI-Red Star’, ‘Argus’, ‘ARTI-Purple’, ‘ARTI-Queen’, and ‘ARTI-Rollypop’, squalene was in the top 10 with high content. dl-α-Tocopherol was also found in all chrysanthemum cultivars except for ‘Argus’, with content range of 2.57% to 0.48% (Table S1). In the case of squalene oxide in the top 10 ranking, it is contained in a high content in group III only. Therefore, the current study showed that the flowers of the novel chrysanthemum mutant cultivars are a rich source of various bioactive phytochemicals.

**Figure 4:**
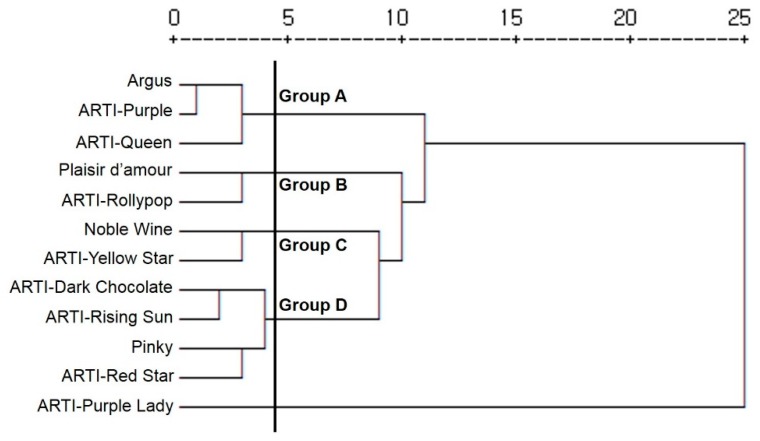
Dendrogram from cluster analysis of volatile components of the 12 chrysanthemum cultivars using the average linkage clustering method.

To compare the volatile compound compositions among the eight chrysanthemum mutant cultivars and the three original cultivars, we performed a cluster analysis based on the contents of the 57 volatile compounds detected by GC-MS analysis (Figure 4). The cluster analysis divided the chrysanthemum cultivars into four clusters and one independent cultivar. Group A consisted of ‘Argus’ and two mutant cultivars (‘ARTI-Purple’ and ‘ARTI-Queen’) derived from ‘Argus’. Group B consisted of ‘ARTI-Rollypop’ and its original cultivar ‘Plaisir d’amour’. Group C contained the mutant cultivar ‘ARTI-Yellow Star’ and the original cultivar ‘Noble Wine’. Group D consisted of ‘Pinky’, two mutant cultivars (‘ARTI-Rising Sun’ and ‘ARTI-Red Star’) derived from ‘Pinky’, and ‘ARTI-Dark Chocolate’. ‘AERI-Purple Lady’ was not placed in a group.

## 3. Materials and Methods

### 3.1. General Procedures

Analytical HPLC-DAD-ESIMS was carried out using an Agilent 1200 series system and an Agilent 6120 quadrupole MS system (Agilent Technologies Co., Santa Clara, CA, USA) equipped with a YMC-Triart C18 column (5 μm, 250 mm × 4.6 mm; YMC Co., Kyoto, Japan) for analysis of flavonoids and phenolic compounds, a YMC-Hydrosphere C18 column (5 μm, 250 mm × 4.6 mm; YMC Co., Kyoto, Japan) for analysis of anthocyanins, and a YMC-Carotenoid column (5 μm, 250 mm × 4.6 mm; YMC Co., Kyoto, Japan) for analysis of carotenoids. Data acquisition and processing were performed using the ChemStation software (Agilent Technologies Co., Santa Clara, CA, USA). Volatile compounds in the flowers of chrysanthemum cultivars were analyzed using a GC-MS (Plus-2010, Shimadzu, Kyoto, Japan) instrument equipped with a DB-5MS column (30 m × 0.25 mm × 0.25 μm; Agilent Technologies Co., Santa Clara, CA, USA). A [^60^Co] γ-irradiator (150 TBq capacity; AECL, Ottawa, Ontario, Canada) was used for gamma-irradiation. The standard compounds apigenin, luteolin, acacetin-7-*O*-β-glucoside, apigenin-7-*O*-β-glucoside, luteolin-7-*O*-β-glucoside, and linarin (acacetin-7-*O*-rutinoside) were isolated from the flowers of ‘ARTI-Dark Chocolate’ as described previously [12]. Chlorogenic acid (Wuhan ChemFaces Biochemical Co., Ltd., Hubei, China), 3,5-dicaffeoylquinic acid (Chengdu Biopurify Phytochemicals Ltd., Chengdu, China), cyanidin chloride (Sigma Chemical Co., St Louis, MO, USA), cyanidin-3-glucoside chloride (Sigma Chemical Co., St Louis, MO, USA), cyanidin 3-*O*-(6ʺ-malonylglucoside) (Cfm Oskar Tropitzsch GmbH, Marktredwitz, Germany), and lutein (Sigma Chemical Co., St Louis, MO, USA) were purchased. All other chemicals and solvents used in the study were of analytical grade.

### 3.2. Plant Material

The radiation mutant cultivars of *Chrysanthemum morifolium* were generated by the treatment of stem cuttings of the original cultivars with various doses of gamma (^60^Co) irradiation as follows: ‘ARTI-Dark Chocolate’ (50 Gy), ‘ARTI-Purple Lady’ (30 Gy), and ‘ARTI-Yellow Star’ (50 Gy) derived from ‘Noble Wine’; ‘ARTI-Red Star’ (50 Gy) and ‘ARTI-Rising Sun’ (30 Gy) derived from ‘Pinky’; ‘ARTI-Purple’ (40 Gy) and ‘ARTI-Queen’ (30 Gy) derived from ‘Argus’; and ‘ARTI-Rollypop’ (70 Gy) derived from ‘Plaisir d’amour’. These mutants were selected from among petal-color variants and exhibited stable inheritance of the petal-color phenotype for four years. The radiation mutant cultivars have been grown by the Radiation Breeding Research Team, Advanced Radiation Technology Institute, Korea Atomic Energy Research Institute and have been registered as new plant varieties in the Korea Seed and Variety Service. These flowers were handpicked and randomly collected at the stage of fully open-flowering in the same plantation. The flowers were freeze-dried, ground to powder, and stored at −20 °C in polyethylene plastic bags until further analysis. Voucher specimens were deposited at the Radiation Breeding Research Center, Advanced Radiation Technology Institute, Korea Atomic Energy Research Institute.

### 3.3. HPLC-DAD-ESIMS and GC-MS Analytical Conditions

The LC-MS analytical conditions for flavonoids, phenolic compounds, anthocyanins, and carotenoids in the flowers were set as follows: positive ionization mode (ESI^+^); scan range = *m/z* 100−1000; scan rate = 1.06 s/cycle; capillary voltage = 4000 V; drying gas flow = 10 L/min (N_2_); nebulizer pressure = 30 psi; drying gas temperature = 350 °C. The GC-MS analytical conditions for volatiles in the flowers were set as follows: ionization voltage, 70 eV; mass scan range, 50–450 mass units; injector temperature, 250 °C; detector temperature, 230 °C; inject volume, 1 μL; splitless; carrier gas, helium; and flow rate, 4.4 mL/min.

### 3.4. Extraction and LC-DAD Analysis of Flavonoids and Phenolic Acids

Dried flowers of the chrysanthemum cultivars were individually ground to powder, and a sample of the powder (1 g for each cultivar) was extracted with 95% ethanol (30 mL) for 24 h at room temperature. The extracted solutions were filtered through a polyvinylidene fluoride syringe filter (0.45 μm) for HPLC analysis. Each extraction was repeated in triplicate. The standard compounds (six flavonoids and two phenolic acids) were separated using a YMC-Triart C18 column (5 μm, 250 mm × 4.6 mm; YMC Co., Kyoto, Japan) equipped with the Agilent 1200 series LC system. Binary gradient elution with 0.1% formic acid in water (*v/v*, solvent A) and 0.1% formic acid in acetonitrile (*v/v*, solvent B) was performed as follows: 0–60 min, 15%–35% B; 60–70 min, 35%–60% B; 70–71 min, 60%–95% B; 71–80 min, 95% B; 80–81 min, 95%–15% B; 81–90 min, 15% B. The total flow rate was maintained at 0.8 mL/min and the injection volume was 10 μL. Chromatograms were acquired at 280 nm by the DAD detector. The standard compounds (six flavonoids and two phenolic acids) were weighed accurately and dissolved in methanol at 1.0 mg/mL. For quantification, calibration curves of these standards were prepared by measurement at four concentrations (25, 50, 75, and 100 μg/mL). The linear equations for chlorogenic acid, luteolin-7-*O*-β-glucoside, 3,5-dicaffeoylquinic acid, apigenin-7-*O*-β-glucoside, linarin (acacetin-7-*O*-rutinoside), acacetin-7-*O*-β-glucoside, luteolin, and apigenin were *y* = 18.675*x* − 17.907, *y* = 14.105*x* − 16.180, *y* = 22.766*x* − 25.32, *y* = 33.317*x* − 9.170, *y* = 21.849*x* − 21.340, *y* = 14.277*x* + 20.120, *y* = 23.094*x* + 5.220, and *y* = 36.953*x* − 46.980, respectively. The calibration curves were linear with high correlation coefficients (*r*^2^ = 0.9991–0.9996).

### 3.5. Extraction and LC-DAD Analysis of Anthocyanins

Samples (each 1 g) of the powder from dried flowers of each chrysanthemum cultivar were extracted with 5% formic acid in water (*v*/*v*, 20 mL) and sonicated for 30 min. Each extracted solution was filtered through a polytetrafluoro-ethylene hydrophilic syringe filter (0.45 μm) for HPLC analysis. Each extraction was repeated in triplicate. Cyanidin chloride, cyanidin-3-glucoside chloride, and cyanidin-3-(6″-malonylglucoside) were separated using a YMC-Hydrosphere C18 column (5 μm, 250 mm × 4.6 mm; YMC Co.) equipped with the Agilent 1200 series LC system. Binary gradient elution with 5% formic acid in water (*v*/*v*, solvent A) and 5% formic acid in acetonitrile (*v*/*v*, solvent B) was performed as follows: 0–40 min, 5%–50% B; 40–41 min, 50%–100% B; 41–45 min, 100% B; 45–46 min, 100%–5% B; 46–50 mi and 5% B. The oven temperature was set to 40 °C. The total flow rate was maintained at 0.8 mL/min and the injection volume was 10 μL. Chromatograms were acquired at 520 nm by the DAD detector. The standard compounds (cyanidin chloride, cyanidin-3-*O*-glucoside chloride, and cyanidin-3-*O*-(6″-malonylglucoside)) were weighed accurately and dissolved in methanol at 1.0 mg/mL. For quantification, calibration curves of these standards were prepared from measurements at four concentrations (25, 50, 75, and 100 μg/mL). The linear equations for cyanidin chloride, cyanidin-3-glucoside chloride, and cyanidin-3-(6″-malonylglucoside) were *y* = 44.603*x*−381.8, *y* = 44.087*x*−134.39, and *y* = 32.221*x*−120.13, respectively. The calibration curves were linear with high correlation coefficients (*r*^2^ = 0.993–0.996).

### 3.6. Extraction and LC-DAD Analysis of Carotenoids

To extract carotenoids from the flowers of the chrysanthemum cultivars, the powdered samples (each 1 g) were extracted with ethanol (20 mL) containing 0.1% ascorbic acid (*w/v*) and the sample tubes were mixed using vortex for 20 s and placed in a water bath at 85 °C for 5 min. The carotenoid extracts were saponified with potassium hydroxide (1.2 mL, 80% *w/v*) at 85 °C for 10 min. After saponification, the sample tubes were placed on ice, and cold deionized water (15 mL) was added. The extracted solutions were filtered through a polyvinylidene fluoride syringe filter (0.45 μm) for HPLC analysis. Each extraction was repeated in triplicate. The standard compounds (lutein and *β*-carotene) were separated using a YMC-Carotenoid column (5 μm, 250 mm × 4.6 mm; YMC Co.) equipped with the Agilent 1200 series LC system. Binary gradient elution with 8% water containing 10 mM ammonium acetate in methanol (*v*/*v*, solvent A) and 100% methyl tert-butyl ether (solvent B) was performed as follows: 0–20 min, 10%–17% B; 20–30 min, 17%–25% B; 30–35 min, 25%–70% B; 35–45 min, 70%–75% B; 45–50 min, 75%–10% B; 50–60 min and 10% B. The total flow rate was maintained at 0.8 mL/min and the injection volume was 10 μL. Chromatograms were acquired at 450 nm by the DAD detector. The standard compound, lutein, was weighed accurately and dissolved in 50% dichloromethane in methanol (*v/v*) at 1.0 mg/mL. For quantification, calibration curves of these standards were prepared from measurements at four concentrations (25, 50, 75, and 100 μg/mL). The linear equation for lutein was *y* = 89.289*x* −221.26. The calibration curves were linear with a high correlation coefficient (*r*^2^ = 0.988). The total content of carotenoids in the 12 chrysanthemum cultivars was calculated from the calibration curve for lutein.

### 3.7. GC-MS Analysis of Volatile Compounds

Extraction of volatile compounds for GC-MS analysis followed the method of a previous study [33] with the following modifications. Fresh flowers were freeze-dried and ground into powder, which was passed through a 40-mesh sieve and hydro-distillation. Flowers of the chrysanthemum cultivars were subjected to hydro-distillation for 4 h with hexane as the collecting solvent, which was dried over anhydrous sodium sulfate to eliminate moisture. The column temperature program specified an isothermal temperature of 40 °C for 5 min followed by an increase to 300 °C at the rate of 5 °C/min and then maintained for 5 min. We identified the substances present in the extracts by retention time and from a mass spectra database (National Institute of Standards and Technologies, Mass Spectra Libraries). The experiment was conducted in triplicate.

### 3.8. Statistical Analysis

Each experiment was done in triplicate and all data are presented as the mean ± standard deviation (SD). The chemical analysis data were subjected to analysis of variance using a multiple comparisons method with the SPSS version 12 statistical software package (SPSS Inc., Chicago, IL, USA). Differences were determined to be significant at α = 0.05. When the treatment effect was significant, means were separated using Duncan’s Multiple Range Test. The resulting matrix of data for volatile compounds was used to construct a dendrogram using the average linkage method with the SPSS version 12 statistical software package.

## 4. Conclusions

In the present study, we obtained abundant information on the phytochemical composition, such as flavonoids, phenolic acids, anthocyanins, carotenoids, and volatile compounds, in the flowers of novel chrysanthemum mutant cultivars. Significant differences in phytochemical composition between the original chrysanthemum cultivars and their γ-irradiated mutant cultivars were observed, depending on the phenotype, such as a change in petal color. The chrysanthemum mutant cultivar which were changed to purple or pink in the color of petals and/or petaloid discs, ‘ARTI-Dark Chocolate’, ‘ARTI-Purple Lady’, ‘ARTI-Purple’, and ‘ARTI-Queen’ had higher total content of flavonoids and phenolic acids than their original cultivars, ‘Noble Wine’ and ‘Argus’. In relation to the phenomenon of petal color variation (red, pink, and/or purple), ‘ARTI-Dark Chocolate’, ‘ARTI-Purple Lady’, ‘ARTI-Red Star’, ‘ARTI-Purple’, ‘ARTI-Queen’, and ‘ARTI-Rollypop’ showed the increase in anthocyanin content. In addition, the total carotenoid content of ‘ARTI-Yellow Star’ with yellow petals was about two-times higher than those of the other cultivars. In the GC analysis of the 12 chrysanthemum cultivars, 57 volatile compounds were identified from five functional groups: hydrocarbons, terpenoids, alcohols, esters, and others. Cluster analysis revealed that the original cultivars clustered closely with the mutant cultivars based on data for the 57 volatile compounds. Thus, we interpret that these volatile compounds are useful for classification and identification of chrysanthemum mutant cultivars. Also, our findings suggest that the novel chrysanthemum mutant cultivars with enhanced content of certain bioactive phytochemicals by radiation breeding may be provided as good source for functional food, pharmaceutical, and horticulture applications.

## Figures and Tables

**Table 1:** The contents of flavonoid and phenolic acid in the chrysanthemum cultivars.

Group	Cultivar	Flavonoids and Phenolic Acids (mg/g, dry weight)								
		Chlorogenic Acid	Luteolin-7-*O*-β-Glucoside	3,5-Dicaffeoyl Quinic Acid	Apigenin-7-*O*-β-Glucoside	Linarin	Acacetin-7-*O*-β-Glucoside	Luteolin	Apigenin	Total
**I**	Noble Wine	0.631 ± 0.025g ^1^	0.875 ± 0.017d	1.198 ± 0.017j	0.218 ± 0.010c	0.135 ± 0.002 b	0.141 ± 0.019 b	0.280 ± 0.006i	0.074 ± 0.010f	3.553 ± 0.105hi
	ARTI-Purple Lady	0.396 ± 0.005j	0.856 ± 0.037d	0.987 ± 0.162k	0.828 ± 0.090 a	0.092 ± 0.002c	0.117 ± 0.014 b	0.467 ± 0.008h	0.081 ± 0.014f	3.824 ± 0.331h
	ARTI-Yellow Star	0.588 ± 0.007h	0.484 ± 0.018e	1.480 ± 0.036i	0.066 ± 0.007e	0.134 ± 0.001 b	0.075 ± 0.008c	0.364 ± 0.032i	0.058 ± 0.002f	3.250 ± 0.111i
II	Pinky	0.976 ± 0.017 b	1.381 ± 0.037 a	4.149 ± 0.066c	0.107 ± 0.022de	ND ^2^d	0.063 ± 0.004c	1.959 ± 0.131c	0.421 ± 0.030d	9.057 ± 0.307 b
	ARTI-Red Star	0.771 ± 0.018f	0.886 ± 0.011d	2.981 ± 0.008g	0.056 ± 0.011e	ND d	0.024 ± 0.004d	2.181 ± 0.119 b	0.430 ± 0.037d	7.329 ± 0.208d
	ARTI-Rising Sun	0.641 ± 0.007g	0.413 ± 0.007e	2.645 ± 0.033h	0.108 ± 0.011de	ND d	0.007 ± 0.001d	1.356 ± 0.020e	1.010 ± 0.009c	6.179 ± 0.088f
III	Argus	0.993 ± 0.010 b	0.115 ± 0.012f	3.397 ± 0.178e	ND f	ND d	ND d	0.738 ± 0.060g	0.343 ± 0.002e	5.588 ± 0.263g
	ARTI-Purple	1.066 ± 0.009 a	0.148 ± 0.015f	3.682 ± 0.066d	0.064 ± 0.014e	ND d	ND d	2.771 ± 0.013 a	3.149 ± 0.014 a	10.880 ± 0.131 a
	ARTI-Queen	0.934 ± 0.007c	0.133 ± 0.016f	4.609 ± 0.094 b	ND f	ND d	0.007 ± 0.001d	0.706 ± 0.084g	0.433 ± 0.002d	6.822 ± 0.204e
IV	Plaisir d’amour	0.797 ± 0.001e	1.030 ± 0.043c	5.855 ± 0.128 a	0.518 ± 0.039 b	ND d	ND d	1.134 ± 0.024f	1.001 ± 0.041c	10.336 ± 0.276 a
	ARTI-Rollypop	0.851 ± 0.015d	0.949 ± 0.079cd	3.463 ± 0.061e	0.126 ± 0.019d	ND d	ND d	1.149 ± 0.010f	1.052 ± 0.027 b	7.589 ± 0.210d

^1^ Mean separation within columns by Duncan’s Multiple Range Tests (*p* ≤ 0.05, *n* = 3); ^2^ not detected.

**Table 2:** Anthocyanin contents of the chrysanthemum cultivars.

Group	Cultivar	Anthocyanins (mg/g, dry weight)			
		Cyanidin-3-*O*-Glucoside	Cyanidin-3-*O*-(6”-Malonyl Glucoside)	Cyanidin	Total
**I**	Noble Wine	0.136 ± 0.002c ^1^	0.754 ± 0.022c	0.348 ± 0.007c	1.239 ± 0.031c
	ARTI-Dark Chocolate	0.545 ± 0.031 a	4.547 ± 0.292 a	1.482 ± 0.095 a	6.574 ± 0.418 a
	ARTI-Purple Lady	0.234 ± 0.001 b	1.558 ± 0.004 b	0.644 ± 0.002 b	2.436 ± 0.008 b
	ARTI-Yellow Star	0.074 ± 0.001ef	0.161 ± 0.007g	0.200 ± 0.002fg	0.434 ± 0.010hi
II	Pinky	0.078 ± 0.001ef	0.192 ± 0.007fg	0.236 ± 0.004ef	0.507 ± 0.012g
	ARTI-Red Star	0.100 ± 0.000d	0.349 ± 0.001de	0.285 ± 0.001de	0.734 ± 0.003ef
	ARTI-Rising Sun	0.067 ± 0.000ef	0.118 ± 0.001g	0.199 ± 0.001fg	0.385 ± 0.002ij
III	Argus	0.065 ± 0.000f	0.096 ± 0.000g	0.177 ± 0.000g	0.339 ± 0.000j
	ARTI-Purple	0.100 ± 0.000d	0.329 ± 0.002ef	0.270 ± 0.001de	0.698 ± 0.004f
	ARTI-Queen	0.082 ± 0.001e	0.189 ± 0.008fg	0.204 ± 0.002fg	0.475 ± 0.012g
IV	Plaisir d’amour	0.100 ± 0.002d	0.377 ± 0.013de	0.301 ± 0.004d	0.778 ± 0.018e
	ARTI-Rollypop	0.113 ± 0.000d	0.496 ± 0.006d	0.281 ± 0.001de	0.890 ± 0.007d

^1^ Mean separation within columns by Duncan’s Multiple Range Tests (*p* ≤ 0.05, *n* = 3).

**Table 3:** Top 10 volatile compounds identified from the chrysanthemum cultivars.

**Group I**				
No.	Noble Wine	ARTI-Dark Chocolate	ARTI-Purple Lady	ARTI-Yellow Star
1	*trans*-3(10)-Caren-2-ol (18.01%)	7-Hexyleicosane (20.23%)	*trans*-3(10)-Caren-2-ol (22.77%)	2-Methylnonadecane (15.65%)
2	2-Methylnonadecane (13.50%)	2-Methylnonadecane (17.79%)	7-Hexyleicosane (10.92%)	7-Hexyleicosane (15.42%)
3	7-Hexyleicosane (12.50%)	9-Octylheptadecane (6.66%)	2-Methylnonadecane (10.37%)	2-Methyleicosane (14.61%)
4	2-Methyleicosane (12.40%)	2-Methyleicosane (6.53%)	2-Methyleicosane (7.24%)	α-2-Dimethyl-2-(4-methyl-3-pentenyl)-cyclopropanemethanol (13.22%)
5	Camphor (9.40%)	α-2-Dimethyl-2-(4-methyl-3-pentenyl)-cyclopropanemethanol (6.36%)	Camphor (7.17%)	Camphor (9.78%)
6	9-Octylheptadecane (4.65%)	3-(1,5-Dimethyl-4-hexenyl)-6-methylenecyclohexene (2.90%)	9-Octylheptadecane (4.50%)	9-Octylheptadecane (5.83%)
7	7,11-Dimethyl-3-methylene-1,6,10-dodecatriene (1.99%)	1-Methyl-5-methylene-8-(1-methylethyl)-1,6-cyclodecadiene (2.43%)	2-Methyltetracosane (3.17%)	dl-α-Tocopherol (2.57%)
8	1-Methyl-5-methylene-8-(1-methylethyl)-1,6-cyclodecadiene (1.96%)	2,2,4-Trimethyl-3-(3,8,12,16-tetramethyl-heptadeca-3,7,11,15-tetraenyl)-cyclohexanol (2.32%)	Squalene (2.64%)	1-Methyl-5-methylene-8-(1-methylethyl)-1,6-cyclodecadiene (2.51%)
9	2-Methyltetracosane (1.77%)	2-Methyltetracosane (2.16%)	1-Methyl-5-methylene-8-(1-methylethyl)-1,6-cyclodecadiene (2.48%)	7,11-Dimethyl-3-methylene-1,6,10-dodecatriene (2.37%)
10	dl-α-Tocopherol (1.65%)	Camphor (1.75%)	7,11-Dimethyl-3-methylene-1,6,10-dodecatriene (1.91%)	Squalene (1.77%)
**Group II**				
No.	Pinky	ARTI-Red Star	ARTI-Rising Sun	
1	2-Methylnonadecane (17.15%)	7-Hexyleicosane (14.21%)	2-Methyl-nonadecane (16.52%)	
2	7-Hexyleicosane (16.86%)	2-Methylnonadecane (13.73%)	7-Hexyleicosane (14.40%)	
3	α-2-Dimethyl-2-(4-methyl-3-pentenyl)-cyclopropanemethanol (12.94%)	α-2-Dimethyl-2-(4-methyl-3-pentenyl)-cyclopropanemethanol (10.20%)	2-Methyl-eicosane (10.40%)	
4	2-Methyltetracosane (6.91%)	Squalene (5.60%)	α-2-Dimethyl-2-(4-methyl-3-pentenyl)-cyclopropanemethanol (8.24%)	
5	9-Octylheptadecane (4.91%)	1-Iodo-2-methylundecane (5.14%)	3,4,5,6-Tetramethyloctane (6.72%)	
6	2-Methyleicosane (4.32%)	2-Methyleicosane (4.30%)	9-Octylheptadecane (4.36%)	
7	9-Octadecenamide (4.26%)	9-Octylheptadecane (3.83%)	2-Methyltetracosane (3.61%)	
8	3-(1,5-Dimethyl-4-hexenyl)-6-methylenecyclohexene (1.93%)	3-(1,5-Dimethyl-4-hexenyl)-6-methylenecyclohexene (3.06%)	2,2,4-Trimethyl-3-(3,8,12,16-tetramethyl-heptadeca-3,7,11,15-tetraenyl)-cyclohexanol (2.71%)	
9	Heptacosane (1.58%)	1-Ethenyl-1-methyl-2,4-bis(1-methylethenyl)cyclohexane (2.78%)	dl-α-Tocopherol (1.71%)	
10	6-Methyltridecane (1.48%)	1-Phenyl-1-nonyne (2.03%)	Heptacosane (1.67%)	
				
**Group III**				
No.	Argus	ARTI-Purple	ARTI-Queen	
1	7-Hexyleicosane (15.10%)	2-Methyleicosane (15.43%),	Squalene oxide (15.38%)	
2	2-Methyleicosane (14.69%)	2-Methylnonadecane (14.87%)	7-Hexyleicosane (12.49%)	
3	2-Methylnonadecane (14.37%)	7-Hexyleicosane (14.10%)	2-Methylnonadecane (11.53%)	
4	9-Octylheptadecane (8.66%)	Squalene oxide (12.29%)	2-Methyleicosane (10.26%)	
5	α-2-Dimethyl-2-(4-methyl-3-pentenyl)-cyclopropanemethanol (7.05%)	9-Octylheptadecane (8.28%)	9-Octylheptadecane (7.76%)	
6	Squalene oxide (6.31%)	α-2-Dimethyl-2-(4-methyl-3-pentenyl)-cyclopropanemethanol (7.20%)	9-(3,3-Dimethyloxiran-2-yl)-2,7-dimethylnona-2,6-dien-1-ol (4.23%)	
7	2-Methyltetracosane (2.59%)	9-(3,3-Dimethyloxiran-2-yl)-2,7-dimethylnona-2,6-dien-1-ol (4.31%)	Squalene (3.82%)	
8	*Z*-12-Pentacosene (1.72%)	1-Octadecanesulphonyl chloride (2.32%)	α-2-Dimethyl-2-(4-methyl-3-pentenyl)-Cyclopropanemethanol (3.77%),	
9	Squalene (1.61%)	Squalene (2.15%)	1-Octadecanesulphonyl chloride (2.03%)	
10	11-decyltetracosane (1.56%)	Heptacosane (1.74%)	*cis*-2-Methyl-7-octadecene (1.68%)	
**Group IV**				
No.	Plaisir d’amour	ARTI-Rollypop		
1	2-Methyl-eicosane (20.00%),	2-Methyl-eicosane (15.22%),		
2	2-Methyl-nonadecane (15.72%)	2-Methyl-nonadecane (13.07%)		
3	7-Hexyleicosane (13.03%)	7-Hexyleicosane (13.07%)		
4	9-Octylheptadecane (4.22%)	9-Octylheptadecane (5.96%)		
5	α-2-Dimethyl-2-(4-methyl-3-pentenyl)-cyclopropanemethanol (2.74%)	2-Methyltetracosane (5.05%)		
6	Bornyl acetate (2.55%)	Bornyl acetate (4.07%)		
7	Heptacosane (2.06%)	Squalene (3.83%)		
8	dl-α-Tocopherol (2.00%)	α-2-Dimethyl-2-(4-methyl-3-pentenyl)-cyclopropanemethanol (3.32%),		
9	1,7,7-Trimethylbicyclo[2.2.1]heptan-2-ol (1.78%)	3-(1,5-Dimethyl-4-hexenyl)-6-methylenecyclohexene (2.23%)		
10	3-(1,5-Dimethyl-4-hexenyl)-6-methylenecyclohexene (1.65%)	1,7,7-Trimethylbicyclo[2.2.1]heptan-2-ol (2.10%)		

## Supplementary Materials

The following are available online: Figure S1: HPLC chromatograms of the flavonoid and phenolic acid profiles of the chrysanthemum cultivars detected at 280 nm. Figure S2: HPLC chromatograms of the anthocyanin profiles of chrysanthemum cultivars detected at 520 nm. Figure S3: HPLC chromatograms of the carotenoid profiles of chrysanthemum cultivars detected at 450 nm. Figure S4: GC-MS chromatograms of the top 10 constituents identified in volatile compounds of the chrysanthemum cultivars. Table S1: Volatile constituents in the flowers of the 12 chrysanthemum cultivars.
