# Supplementary material for: Comparative Analysis of Phytochemical Composition of Gamma-Irradiated Mutant Cultivars of Chrysanthemum morifolium
Source: Molecules. 2019 Aug 19;24(16):3003. doi: 10.3390/molecules24163003 (PMC6720760; doi:10.3390/molecules24163003)
Supplement: Supplementary file 1 [file molecules-24-03003-s001.pdf]

Supplementary Materials

Comparative Analysis of Phytochemical Composition of Gamma-Irradiated Mutant Cultivars of *Chrysanthemum morifolium*

Jaihyunk Ryu, Bomi Nam, Bo Ram Kim, Sang Hoon Kim, Yeong Deuk Jo, Joon-Woo Ahn, Jin-Baek Kim, Chang Hyun Jin and Ah-Reum Han *

Advanced Radiation Technology Institute, Korea Atomic Energy Research Institute, Jeongeup-si, Jeollabuk-do 56212, Republic of Korea; jhryu@kaeri.re.kr (J.R.); bomi1201@kaeri.re.kr (B.N.); boram1606@kaeri.re.kr (B.R.K); shkim80@kaeri.re.kr (S.H.K.); jyd@kaeri.re.kr (Y.D.J.); joon@kaeri.re.kr (J.-W.A); jbkim74@kaeri.re.kr (J.-B.K.); chjin@kaeri.re.kr (C.H.J.); arhan@kaeri.re.kr (A.-R.H.)

* Correspondence: arhan@kaeri.re.kr; Tel.: +82-63-570-3167 (A.-R.H.)

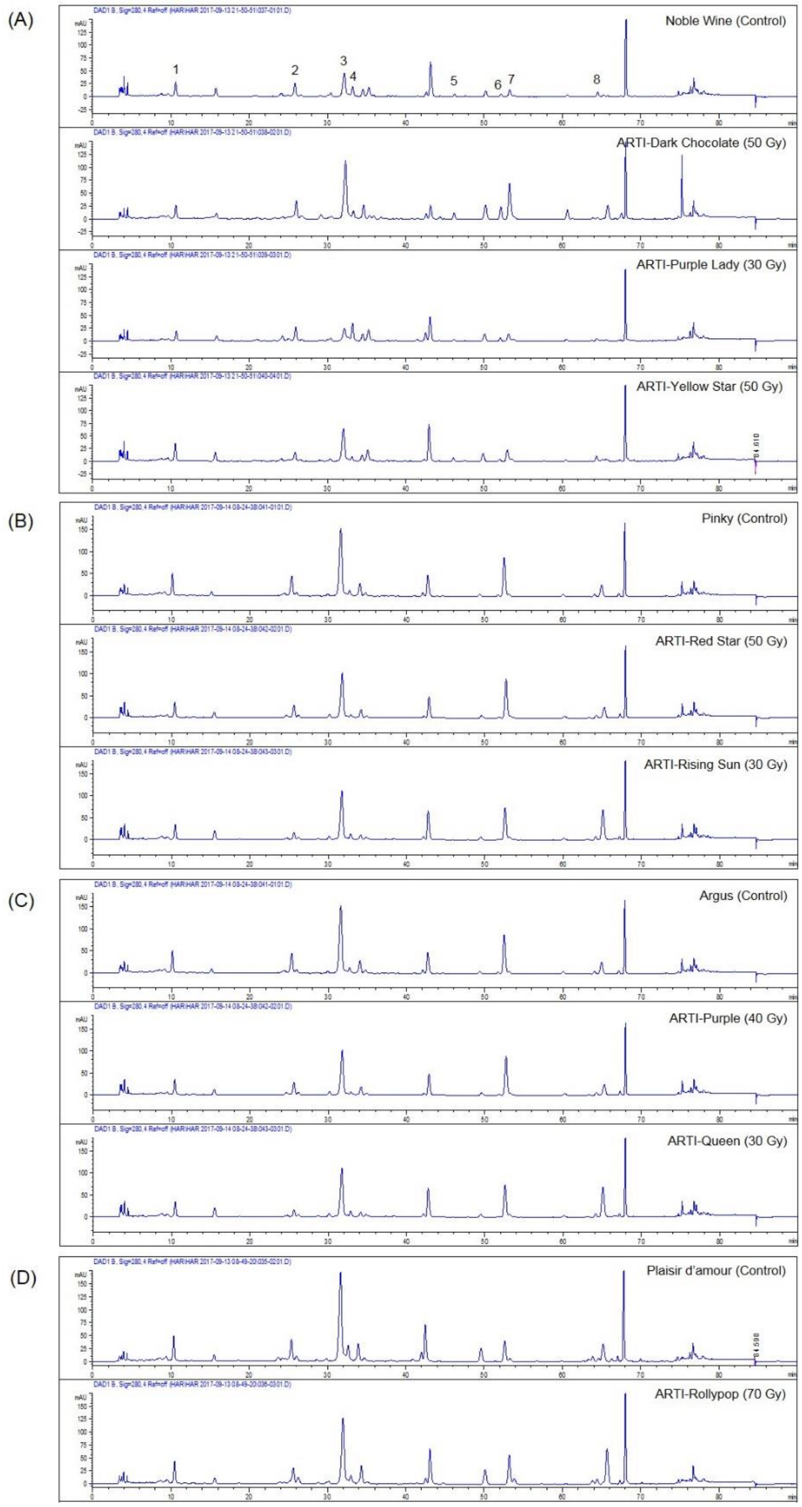

Figure S1. HPLC chromatograms of the flavonoid and phenolic acid profiles of *Chrysanthemum* cultivars detected at 280 nm. (A) The original cultivar 'Noble Wine' and its γ-irradiated mutant lines 'ARTI-Dark Chocolate' (50 Gy), 'ARTI-Dark Chocolate' (50 Gy), 'ARTI-Purple Lady' (30 Gy), and 'ARTI-Yellow Star' (50 Gy); (B) the original cultivar 'Pinky' and its γ-irradiated mutant lines 'ARTI-Red Star' (50 Gy) and 'ARTI-Rising Sun' (30 Gy); (C) the original cultivar 'Argus' and its γ-irradiated mutant lines 'ARTI-Purple' (40 Gy) and 'ARTI-Queen' (30 Gy); (D) the original cultivar 'Plaisir d'amour' and its γ-irradiated mutant line 'ARTI-Rollypop' (70 Gy). Peak identity: 1, chlorogenic acid; 2, luteolin-7-*O*-β-glucoside; 3, 3,5-dicaffeoylquinic acid; 4, apigenin-7-*O*-β-glucoside; 5, linarin; 6, acacetin-7-*O*-β-glucoside; 7, luteolin; 8, apigenin.

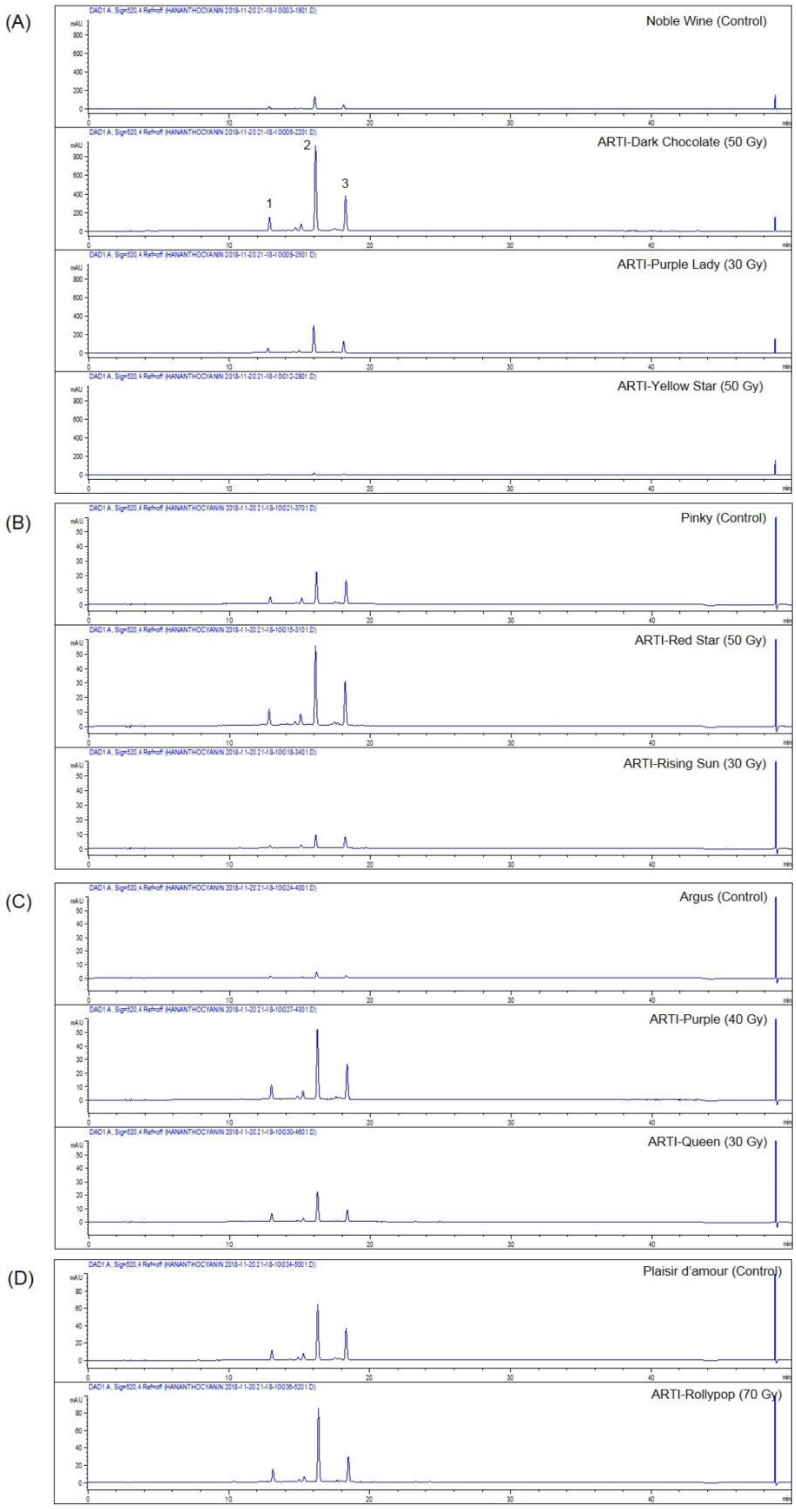

Figure S2. HPLC chromatograms of the anthocyanin profiles of *Chrysanthemum* cultivars detected at 520 nm. (A) The original cultivar 'Noble Wine' and its γ-irradiated mutant lines 'ARTI-Dark Chocolate' (50 Gy), 'ARTI-Dark Chocolate' (50 Gy), 'ARTI-Purple Lady' (30 Gy), and 'ARTI-Yellow Star' (50 Gy); (B) the original cultivar 'Pinky' and its γ-irradiated mutant lines 'ARTI-Red Star' (50 Gy) and 'ARTI-Rising Sun' (30 Gy); (C) the original cultivar 'Argus' and its γ-irradiated mutant lines 'ARTI-Purple' (40 Gy) and 'ARTI-Queen' (30 Gy); (D) the original cultivar 'Plaisir d'amour' and its γ-irradiated mutant line 'ARTI-Rollypop' (70 Gy). Peak identity: 1, cyanidin-3-*O*-glucoside; 2, cyanidin-3-*O*-(6″-malonylglucoside); 3, cyanidin.

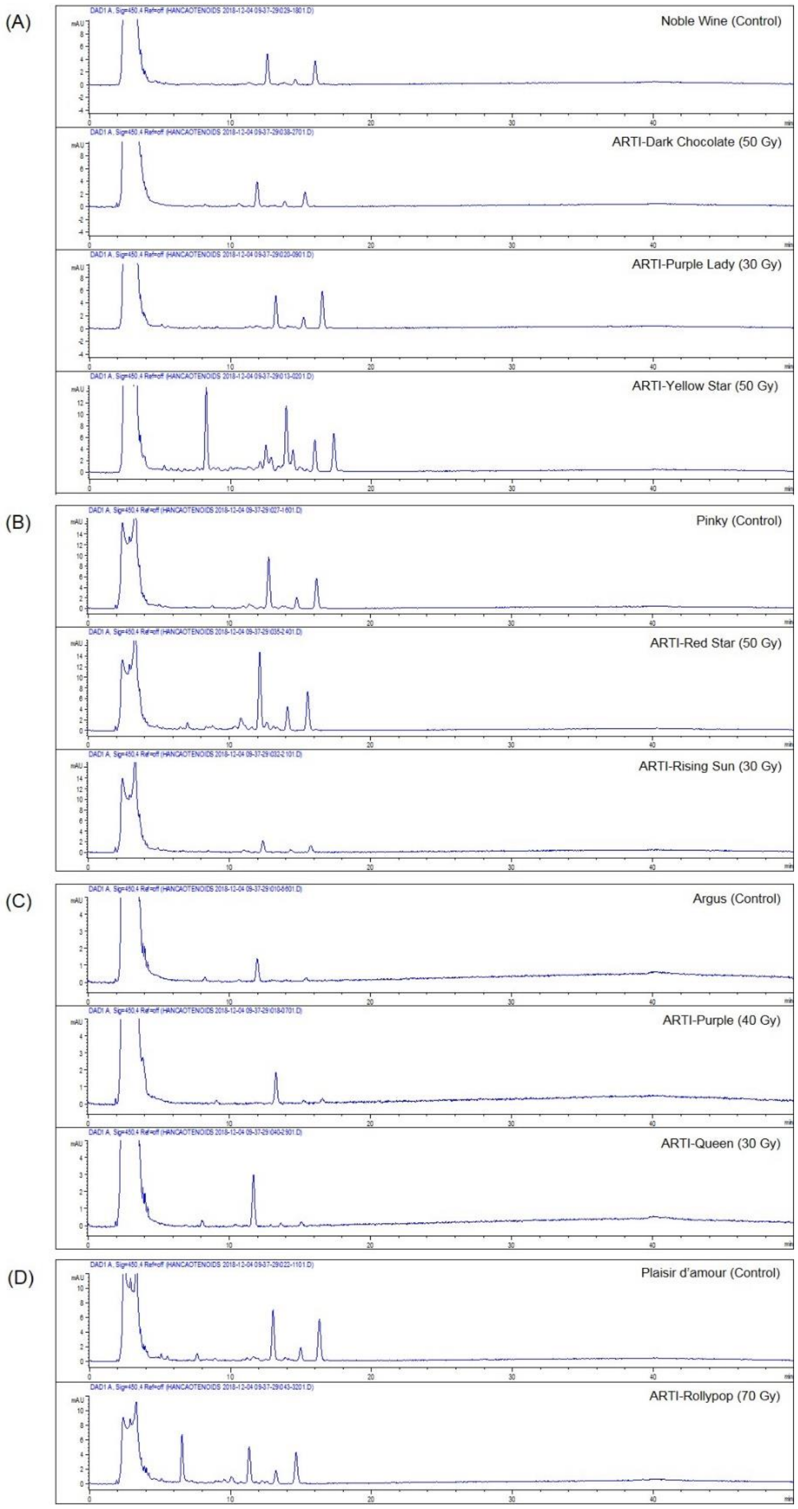

Figure S3. HPLC chromatograms of the cartotenoid profiles of *Chrysanthemum* cultivars detected at 450 nm. (A) The original cultivar 'Noble Wine' and its γ-irradiated mutant lines 'ARTI-Dark Chocolate' (50 Gy), 'ARTI-Dark Chocolate' (50 Gy), 'ARTI-Purple Lady' (30 Gy), and 'ARTI-Yellow Star' (50 Gy); (B) the original cultivar 'Pinky' and its γ-irradiated mutant lines 'ARTI-Red Star' (50 Gy) and 'ARTI-Rising Sun' (30 Gy); (C) the original cultivar 'Argus' and its γ-irradiated mutant lines 'ARTI-Purple' (40 Gy) and 'ARTI-Queen' (30 Gy); (D) the original cultivar 'Plaisir d'amour' and its γ-irradiated mutant line 'ARTI-Rollypop' (70 Gy).

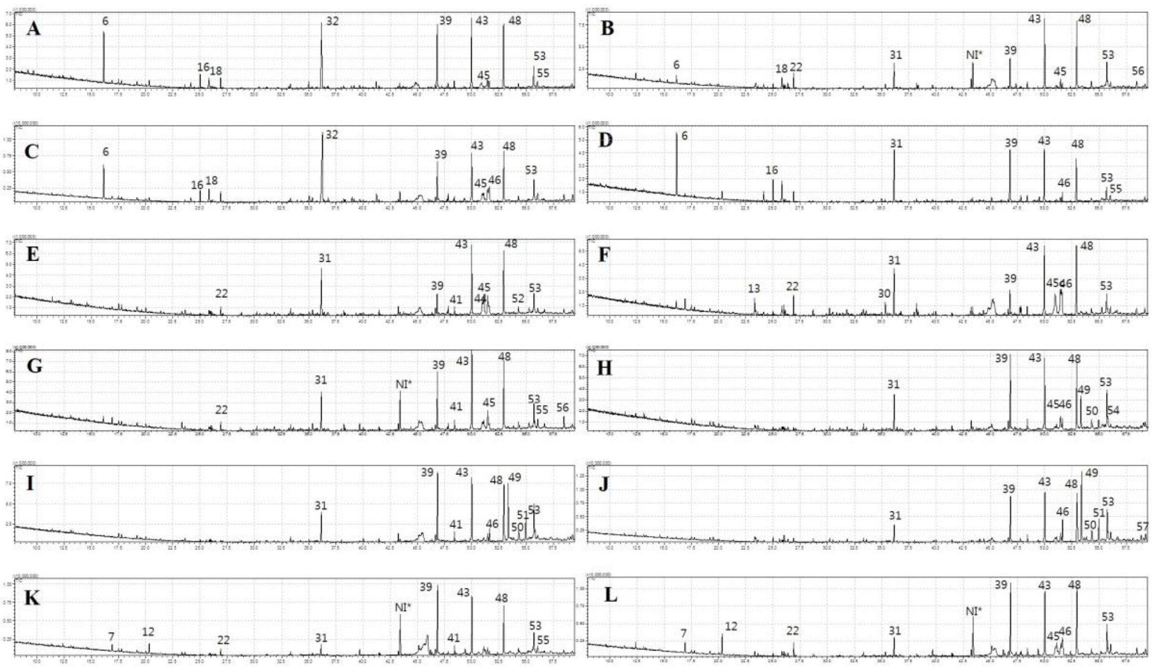

Figure S4. GC-MS chromatograms of the top ten constituents identified in volatile compounds of the *Chrysanthemum* cultivars. (A) 'Noble Wine' (control); (B) 'ARTI-Dark Chocolate' (50 Gy); (C) 'ARTI-Purple Lady' (30 Gy); (D) 'ARTI-Yellow Star' (50 Gy); (E) 'Pinky' (control); (F) 'ARTI-Red Star' (50 Gy); (G) 'ARTI-Rising Sun' (30 Gy); (H) 'Argus' (control); (I) 'ARTI-Purple' (40 Gy); (J) 'ARTI-Queen' (30 Gy); (K) 'Plaisir d'amour' (control); (L) 'ARTI-Lollipop' (70 Gy). Peak identity: 6, Camphor; 7, 1,7,7-Trimethylbicyclo[2.2.1]heptan-2-ol; 12, Bornyl acetate; 13, 1-Ethenyl-1-methyl-2,4-bis(1-methylethenyl)cyclohexane; 16, 7,11-Dimethyl-3-methylene-1,6,10-dodecatriene; 18, 1-Methyl-5-methylene-8-(1-methylethyl)-1,6-cyclodecadiene; 22, 3-(1,5-Dimethyl-4-hexenyl)-6-methylenecyclohexene; 30, 1-Phenyl-1-nonyne; 31, α-2-Dimethyl-2-(4-methyl-3-pentenyl)-cyclopropanemethanol; 32, *trans*-3(10)-Caren-2-ol; 39, 2-Methyleicosane; 41, Heptacosane; 43, 2-Methyl-nonadecane; 45, 2-Methyltetracosane; 46, Squalene; 48, 7-Hexyleicosane; 49, Squalene oxide; 50, 11-Decyltetracosane; 51, 9-(3,3-Dimethyloxiran-2-yl)-2,7-dimethylnona-2,6-dien-1-ol; 53, 9-Octylheptadecane; 54, *Z*-12-Pentacosene; 55, DL-α-tocopherol; 56, 2,2,4-Trimethyl-3-(3,8,12,16-tetramethyl-heptadeca-3,7,11,15-tetraenyl)-cyclohexanol; 57, *cis*-2-Methyl-7-octadecene; NI, not identified.

Table S1. Volatile constituents in the flowers of the twelve chrysanthemum cultivars.

No.	Retention time	Compound names	Molecular Formula	Similarity (%)	Group I								Group II						Group III						Group Ⅵ			
					Noble Wine		ARTI-Dark Chocolate		ARTI-Purple Lady		ARTI-Yellow Star		Pinky		ARTI-Red Star		ARTI-Rising Sun		Argus		ARTI-Purple		ARTI-Queen		Plaisir d'amour		ARTI-Rollipop	
1	11.37	2,4-Dimethylhexane	C_8H_{18}	91	0.38	bc	0.16	cd	0.28	bc	0.00	d	0.00	d	0.00	d	0.00	d	0.83	a	0.22	cd	0.42	b	0.00	cd	0.10	cd
2	11.40	Decane	$C_{10}H_{22}$	93	0.00	b	0.15	b	0.00	b	0.00	b	0.41	a	0.00	b	0.00	b	0.00	b	0.51	a	0.00	b	0.00	b	0.00	b
3	12.38	Eucalyptol	$C_{10}H_{18}O$	91	0.46	b	1.16	a	0.00	c	0.43	b	0.00	c	0.00	c	0.00	c	0.39	b	0.20	c	0.60	b	0.58	b	1.09	a
4	13.32	4-Methyl-1-undecene	$C_{12}H_{24}$	90	0.00	b	0.15	b	0.21	b	0.00	b	0.84	a	0.00	b	0.00	b	0.00	b	0.18	b	0.00	b	0.00	b	0.00	b
5	15.30	2,6-Dimethyl-3,5-heptadien-2-ol	$C_9H_{16}O$	93	0.00	c	0.55	a	0.00	c	0.00	c	0.00	c	0.00	c	0.00	c	0.00	c	0.00	c	0.41	b	0.00	c	0.00	c
6	16.13	Camphor	$C_{10}H_{16}O$	92	9.40	a	1.75	b	7.17	a	9.78	a	0.00	c	1.09	b	1.15	b	0.41	c	0.00	c	0.27	c	0.00	c	0.00	c
7	16.92	1,7,7-Trimethylbicyclo[2.2.1]heptan-2-ol	$C_{10}H_{18}O$	91	0.29	c	0.00	d	0.39	c	0.00	d	0.00	d	1.60	b	1.50	b	0.00	d	0.00	d	0.00	d	1.78	b	2.10	a
8	17.51	Methyl salicylate	$C_8H_8O_3$	91	0.68	cd	0.48	de	0.60	cd	0.00	f	1.42	a	0.34	e	0.76	bc	1.23	a	0.95	b	0.44	de	0.86	bc	0.56	cd
9	19.22	1,3-Bis(1,1-dimethylethyl)benzene	$C_{14}H_{22}$	90	0.50	bc	0.17	cd	0.18	cd	0.00	d	1.17	a	0.00	d	0.60	b	1.08	a	0.45	bc	0.50	bc	0.62	bc	0.53	bc
10	19.51	2,6,6-Trimethyl-bicyclo[3.1.1]hept-2-en-4-ol acetate	$C_{12}H_{20}O_3$	92	0.00	b	0.00	b	0.00	b	0.00	b	0.00	b	0.00	b	0.00	b	0.90	a	0.00	b	0.83	a	0.00	b	0.00	b
11	20.02	2,6-Methyldecane	$C_{12}H_{26}$	90	0.15	de	0.17	de	0.17	de	0.00	e	0.89	a	0.00	e	0.57	bc	0.82	ab	0.35	cd	0.20	de	0.71	c	0.25	de
12	20.32	Bornyl acetate	$C_{12}H_{20}O_2$	91	0.49	c	0.00	d	0.20	d	0.85	c	0.00	d	0.00	d	0.00	d	0.00	d	0.18	d	0.00	d	2.55	b	4.07	a
13	23.33	1-Ethenyl-1-methyl-2,4-bis(1-methylethenyl)cyclohexane	$C_{15}H_{24}$	92	0.42	d	0.00	f	0.28	ef	0.00	f	0.51	de	2.78	a	1.11	b	0.62	cd	0.00	f	0.91	bc	0.83	cd	0.74	cd
14	23.62	4,8-Dimethyltridecane	$C_{15}H_{32}$	90	0.43	d	0.21	e	0.28	de	0.00	f	1.01	a	0.00	f	0.60	c	0.81	b	0.61	c	0.30	de	0.51	d	0.39	d
15	24.17	*cis*-α-Bisabolene	$C_{15}H_{24}$	90	1.00	a	0.65	ab	1.06	a	1.27	a	0.00	b	0.00	b	0.00	b	0.00	b	0.00	b	0.00	b	0.00	b	0.00	b
16	25.02	7,11-Dimethyl-3-methylene-1,6,10-dodecatriene	$C_{15}H_{24}$	93	1.99	a	0.76	b	1.91	a	2.37	a	0.00	b	0.21	b	0.00	b	0.00	b	0.24	b	0.81	b	0.00	b	0.34	b
17	25.60	Caryophyllene	$C_{15}H_{24}$	92	0.00	c	0.00	c	0.00	c	0.00	c	0.00	c	0.00	c	0.00	c	0.00	c	0.33	b	0.69	a	0.00	c	0.00	c
18	25.82	1-Methyl-5-methylene-8-(1-methylethyl)-1,6-cyclodecadiene	$C_{15}H_{24}$	90	1.96	ab	2.43	ab	2.48	a	2.51	a	1.02	cd	1.35	bc	0.00	e	0.68	de	0.00	e	0.28	de	0.00	e	0.78	de
19	25.93	2,3,6,7-Tetramethyl-octane	$C_{12}H_{26}$	90	0.28	c	0.31	c	0.00	d	0.00	d	0.94	a	0.00	d	0.00	d	0.84	ab	0.46	bc	0.23	c	1.10	ab	0.37	c
20	26.02	(*Z,Z*)-α-Farnesene	$C_{15}H_{24}$	90	0.00	e	0.84	c	0.27	d	0.28	d	0.73	c	1.47	a	0.85	c	0.26	d	0.35	d	1.23	b	0.00	e	0.63	c
21	26.38	α-Farnesene	$C_{15}H_{24}$	91	0.00	d	0.93	a	0.00	d	0.00	d	0.00	d	0.00	d	0.00	d	0.00	d	0.00	d	0.59	b	0.00	d	0.42	c
22	26.91	3-(1,5-Dimethyl-4-hexenyl)-6-methylenecyclohexene	$C_{15}H_{24}$	90	1.52	de	2.90	ab	1.71	cd	1.07	de	1.93	c	3.06	a	1.55	de	0.63	f	0.00	g	0.88	ef	1.65	de	2.23	bc
23	28.70	*trans*-3,6-Diethyl-3,6-dimethyltricyclo[3.1.0.0(2,4)]hexane	$C_{12}H_{20}$	91	0.00	e	0.34	d	0.00	e	0.00	e	0.00	e	0.72	a	0.49	c	0.00	e	0.00	e	0.62	b	0.00	e	0.36	d
24	30.22	Octahydro-1,4,9,9-tetramethyl-1*H*-3a,7-methanoazulene	$C_{15}H_{26}$	90	0.00	d	0.00	d	0.00	d	0.00	d	0.76	c	1.28	a	0.78	bc	0.70	d	0.00	d	0.83	b	0.00	d	0.00	d
25	33.25	9,12-Octadecadienoyl chloride	$C_{18}H_{31}ClO$	91	0.39	a	0.00	b	0.00	b	0.34	a	0.00	b	0.00	b	0.00	b	0.00	b	0.00	b	0.00	b	0.00	b	0.00	b
26	33.33	1-Octadecene	$C_{18}H_{36}$	92	0.79	cd	0.54	de	0.54	de	0.30	e	1.32	a	0.96	bc	1.17	ab	1.15	ab	0.74	cd	0.66	d	0.78	cd	0.51	de
27	33.48	2,6,11-Trimethyldodecan,	$C_{15}H_{32}$	90	0.00	b	0.00	b	0.00	b	0.00	b	0.28	a	0.00	b	0.00	b	0.00	b	0.00	b	0.00	b	0.00	b	0.00	b
28	34.24	Cyclopentadecanol	$C_{15}H_{30}O$	92	0.00	b	0.00	b	0.00	b	0.28	a	0.00	b	0.00	b	0.00	b	0.00	b	0.00	b	0.00	b	0.00	b	0.00	b
29	35.00	α-Bisabolol	$C_{15}H_{26}O$	90	1.22	a	0.00	c	1.24	a	0.73	b	0.92	b	0.00	c	0.00	c	0.00	c	0.20	c	0.00	c	0.00	c	0.00	c
30	35.34	1-Phenyl-1-nonyne	$C_{15}H_{20}$	91	0.19	d	0.88	b	0.56	c	0.00	d	0.90	b	2.03	a	0.86	a	0.00	d	0.00	d	0.00	d	0.00	d	0.58	c
31	36.19	α-2-Dimethyl-2-(4-methyl-3-pentenyl)-cyclopropanemethanol	$C_{12}H_{22}O$	90	0.00	ab	6.36	bc	0.00	f	13.22	a	12.94	bc	10.20	ab	8.24	ab	7.05	ab	7.20	ab	3.77	cd	2.74	e	3.32	cd
32	36.24	*trans*-3(10)-Caren-2-ol	$C_{10}H_{16}O$	92	18.01	b	0.00	c	22.77	a	0.00	c	0.00	c	0.00	c	0.00	c	0.00	c	0.00	c	0.00	c	0.00	c	0.00	c
33	39.69	Octadecane	$C_{18}H_{38}$	90	0.00	e	0.72	c	0.26	d	0.00	e	0.00	e	0.00	e	1.07	a	0.00	e	0.00	e	0.00	e	1.31	a	0.88	b
34	39.81	2,6-Dimethyl-1,7-octadien-3-ol	$C_{10}H_{18}O$	92	0.00	b	0.00	b	0.00	b	0.00	b	0.00	b	0.54	a	0.00	b	0.00	b	0.00	b	0.00	b	0.00	b	0.00	b
35	41.46	Cycloeicosane	$C_{20}H_{40}$	90	0.21	d	0.37	cd	0.18	d	0.00	e	0.90	a	0.23	de	0.49	bc	0.66	b	0.62	bc	0.41	cd	0.58	bc	0.19	d
36	41.56	2,3,3-Trimethyl-octane	$C_{11}H_{24}$	91	0.00	b	0.00	b	0.00	b	0.00	b	0.00	b	0.00	b	0.00	b	0.00	b	0.00	b	0.00	b	0.00	a	0.13	a
37	43.38	2-Methyleicosane	$C_{21}H_{44}$	95	0.00	b	0.00	b	0.00	b	0.00	b	0.00	b	0.41	a	0.00	b	0.00	b	0.00	b	0.59	a	0.00	b	0.00	b
38	46.19	2-Methyltricosane	$C_{24}H_{50}$	93	0.00	c	0.00	c	0.00	c	0.00	c	0.00	c	0.00	c	0.00	c	0.00	c	0.00	c	0.00	c	0.87	a	0.82	b
39	46.80	2-Methyleicosane	$C_{21}H_{44}$	96	12.40	c	6.53	e	7.24	e	14.61	b	4.32	f	4.30	f	10.40	d	14.69	b	15.43	b	10.26	d	22.34	a	15.22	b
40	47.24	Diisooctyl phthalate	$C_{24}H_{38}O_4$	90	0.30	a	0.00	b	0.00	b	0.00	b	0.00	b	0.00	b	0.00	b	0.00	b	0.00	b	0.00	b	0.00	b	0.00	b
41	48.41	Heptacosane	$C_{27}H_{56}$	91	0.72	bc	1.34	bc	0.50	c	0.99	bc	1.58	bc	0.67	c	1.67	bc	0.88	bc	1.74	a	1.24	bc	2.06	a	1.12	bc
42	49.38	2,21-Dimethyldocosane	$C_{24}H_{50}$	90	0.63	ab	0.32	b	0.62	b	0.75	ab	0.00	c	0.67	ab	0.00	c	0.87	ab	0.18	b	0.18	b	1.10	a	0.91	ab
43	50.00	2-Methylnonadecane	$C_{20}H_{42}$	95	13.50	cd	17.79	a	10.37	e	15.65	ab	17.15	a	13.73	cd	16.52	ab	14.37	bc	14.87	bc	11.53	d	17.62	ab	13.07	cd
44	51.10	9-Octadecenamide	$C_{18}H_{35}NO$	91	0.00	c	0.35	c	1.36	b	0.00	c	4.26	a	0.00	c	1.48	b	0.78	b	0.00	c	0.68	b	0.00	b	1.43	b

45	51.45	2-Methyltetracosane	$C_{25}H_{52}$	90	1.77	d	2.16	cd	3.17	cd	1.47	d	6.91	a	5.14	b	3.61	bc	2.59	cd	1.38	d	1.47	d	1.45	d	5.05	b
46	51.61	Squalene	$C_{30}H_{50}$	91	1.34	cd	1.28	cd	2.64	bc	1.59	cd	0.00	e	5.60	a	0.78	d	1.61	cd	2.15	bc	3.82	ab	0.50	d	3.83	ab
47	52.37	2,21-Dimethyldocosane	$C_{24}H_{50}$	90	0.00	d	0.00	d	0.00	d	0.14	c	0.00	d	0.00	d	0.00	d	0.00	d	0.00	d	0.00	d	0.48	b	0.62	a
48	52.94	7-Hexyleicosane	$C_{26}H_{54}$	95	12.50	de	20.23	a	10.92	de	15.42	bc	16.86	b	14.21	cd	14.40	cd	15.10	bc	14.10	cd	12.49	de	14.54	cd	13.07	cd
49	53.34	Squalene oxide	$C_{30}H_{50}$	91	0.00	d	0.00	d	0.00	d	0.00	d	0.00	d	0.00	d	0.00	d	6.31	c	12.29	b	15.38	a	0.00	d	0.00	d
50	54.96	11-decyltetracosane	$C_{34}H_{70}$	92	0.82	de	1.37	bc	0.78	ef	0.93	de	1.13	cd	0.98	de	1.18	cd	1.56	b	2.32	a	2.03	a	0.64	ef	0.71	ef
51	54.96	9-(3,3-Dimethyloxiran-2-yl)-2,7-dimethylnona-2,6-dien-1-ol	$C_{15}H_{26}O_2$	90	0.00	c	0.00	c	0.00	c	0.00	c	0.00	c	0.00	c	0.00	c	1.48	b	4.31	a	4.23	a	0.00	c	0.00	c
52	55.27	6-methyltridecane	$C_{14}H_{30}$	92	0.00	d	0.00	d	0.00	d	0.00	d	1.48	a	0.95	b	0.95	b	0.00	d	0.00	d	0.00	d	0.00	d	0.47	c
53	55.71	9-octylheptadecane	$C_{25}H_{52}$	94	4.65	d	6.66	b	4.50	d	5.83	bc	4.91	cd	3.83	d	4.36	d	8.66	a	8.28	a	7.76	a	4.22	cd	5.96	bc
54	55.80	*Z*-12-Pentacosene	$C_{25}H_{50}$	90	0.00	c	1.37	a	0.00	c	0.00	c	0.00	c	0.60	bc	0.56	bc	1.72	a	1.61	a	0.72	b	0.00	c	0.39	bc
55	56.56	DL-α-tocopherol	$C_{29}H_{50}O_2$	90	1.65	b	1.48	b	1.88	ab	2.57	a	0.73	cd	1.19	bc	1.71	b	0.00	e	0.48	de	1.67	b	2.00	ab	1.94	ab
56	58.48	2,2,4-Trimethyl-3-(3,8,12,16-tetramethyl-heptadeca-3,7,11,15-tetraenyl)-cyclohexanol	$C_{30}H_{52}O$	91	0.00	d	2.32	a	1.34	b	0.00	d	0.57	c	1.39	b	2.71	a	0.00	d	0.00	d	0.00	d	0.60	c	1.24	b
57	59.23	*cis*-2-Methyl-7-octadecene	$C_{19}H_{38}$	92	0.51	b	0.62	b	1.54	ab	1.10	ab	0.63	b	0.79	ab	0.00	c	1.13	ab	0.00	c	1.68	a	0.00	c	0.65	b
		Total			91.55		86.8		89.6		94.48		89.42		82.32		82.23		90.81		92.93		91.61		85.02		87.07	

[1] Mean separation within columns by Ducan's Multiple Range Tests ($p \le 0.05$)
